# Supplementary material for: The Effects of the Combined Co-Expression of GroEL/ES and Trigger Factor Chaperones on Orthopoxvirus Phospholipase F13 Production in E. coli
Source: BioTech (Basel). 2024 Dec 23;13(4):57. doi: 10.3390/biotech13040057 (PMC11674265; doi:10.3390/biotech13040057)
Supplement: Supplementary file 1 [file biotech-13-00057-s001.zip › biotech-3347325-supplementary.pdf]

The Effects of the Combined Co-Expression of GroEL/ES and Trigger Factor Chaperones on Orthopoxvirus Phospholipase F13 Production in *E. coli*

Iuliia A. Merkuleva, Vladimir N. Nikitin, Tatyana D. Belaya, Egor. A. Mustaev and Dmitriy N. Shcherbakov

```
1   MSPILGYWKI KGLVQPTRLL LEYLEEKYEE HLYERDEGDK WRNKKFELGL EFPNLPYYID GDVKLTQSMA
71  IIRYIADKHN MLGGCPKERA EISMLEGAVL DIRYGVSRIA YSKDFETLKV DFLSKLPEML KMFEDRLCHK
141 TYLNGDHVTH PDFMLYDALD VVLYMDPMCL DAFPKLVCFK KRIEAIPQID KYLKSSKYIA WPLQGWQATF
211 GGGDHPPKGS TSAVLQSGMW PFASVPAGAK CRLVETLPEN MDFRSDHLTT FECFNEIITL AKKYIYIASF
281 CCNPLSTTRG ALIFDKLKEA SEKGIKIIVL LDERGKRNLG ELQSHCPDIN FITVNIDKKN NVGLLLGCFW
351 VSDDERCYVG NASFTGGSIH TIKTLGVYSD YPPLATDLRR RFDTFKAFNS AKNSWLNLCS AACCLPVSTA
421 YHIKNPIGGV FFTDSPEHLL GYSRDLDTDV VIDKLKSAKT SIDIEHLAIV PTTRVDGNSY YWPDIYNSII
491 EAAINRGVKI RLLVGNWDKN DVYSMATARS LDALCVQNDL SVKVFTIQNN TKLLIVDDEY VHITSANFDG
561 THYQNHGFVS FNSIDKQLVS EAKKIFERDW VSSHSKSLKI HHHHHH*
```

Figure S1. Amino acid sequence of GST-F13 protein. The F13 sequence is highlighted
